# Challenges and Opportunities for Lung Ultrasound in Novel Coronavirus Disease (COVID-19)

**DOI:** 10.4269/ajtmh.20-0323

**Published:** 2020-04-24

**Authors:** Marcus J. Schultz, Chaisith Sivakorn, Arjen M. Dondorp

**Affiliations:** 1 Mahidol–Oxford Tropical Medicine Research Unit (MORU), Mahidol University, Bangkok, Thailand;; 2 Nuffield Department of Medicine, Mahidol University, Bangkok, Thailand;; 3 Department of Intensive Care, Amsterdam University Medical Centers, Amsterdam, The Netherlands;; 4 Department of Clinical Tropical Medicine, Faculty of Tropical Medicine, Mahidol University, Bangkok, Thailand

Novel coronavirus disease (COVID-19) is responsible for hundreds of thousands of hospitalizations worldwide, and this number is still increasing rapidly in many countries as of mid-April 2020. Diagnostic and clinical approaches differ between countries and even within countries because of inconsistent presence of testing materials and variable access to chest imaging. Severe acute respiratory syndrome-coronavirus-2 reverse transcriptase–polymerase chain reaction from nasopharyngeal or throat swabs has a high specificity but moderate sensitivity. Although patients with COVID-19 can present with a normal chest X-ray early in the disease,^1^ abnormalities on chest images have a higher sensitivity to detect the disease than molecular testing, but lack specificity.^2^ Typical findings on a chest X-ray include bilateral and multi-lobar infiltrates that can progress rapidly over the first days of illness. Chest X-ray is the most commonly used imaging technique in suspected COVID-19 in resource-limited settings. However, the image quality is often poor, and follow-up chest X-rays are difficult in settings with constraints on infrastructure, human resources, and personal protective equipment.

Chest computed tomography (CT) scan is more sensitive that chest X-ray to detect early COVID-19. Typical findings on chest CT include bilateral and multi-lobar “ground-glass” or “crazy-paving” opacities, surrounded by spared lung tissue.^3,4^ Ground-glass and crazy-paving opacities can, however, also be seen in many other pulmonary diseases. The COVID-19 working group of the Dutch Radiological Society recently coined the “CO-RADS classification” for diagnosing COVID-19, grading the level of suspicion for COVID-19 from very low (“CO-RADS 1”) to very high (“CO-RADS 5”).^5^ Chest CT scan findings, however, are of limited value in resource-limited settings where access to CT scanners is in general very limited. Chest CT scan access may even be limited in resource-rich settings when hospital systems are overwhelmed with huge numbers of patients with suspected COVID-19. In addition, obtaining a chest CT scan is highly impractical once a patient is intubated and ventilated because transportation to the CT scanner is labor intensive and not without risk. Last, but not least, an additional burden is that the CT scanner needs to be thoroughly cleaned after each suspected case of COVID-19, to prevent the spread of the infection to other patients and healthcare personnel.

Point-of-care lung ultrasound (LUS) could play an important role in COVID-19 management. Lung ultrasound is a noninvasive, rapid, repeatable, and sensitive bedside method to detect a range of pulmonary (and extrapulmonary) pathologies, including pneumothorax, pleural effusions, pulmonary infiltrates, and lung consolidations. In this issue of the *American Journal of Tropical Medicine and Hygiene*, Yasukawa and Taro^6^ report their first experience with LUS in a case series of COVID-19 patients in the United States. All patients had “comet tails” (or “glass rockets”), with or without a “white lung” (“Birolleau variant”), and confluent B lines. Irregular pleural lines were also present in all patients, and half of the patients had small subpleural consolidations. In the one patient with a concomitant chest CT scan, peripheral opacification on the CT scan matched with a white lung area on the LUS images.

The good sensitivity of bedside LUS to detect lung lesions in COVID-19 makes this an attractive additional diagnostic tool in patients suspected of COVID-19, often nursed in isolation rooms or on isolation wards. Lung ultrasound has been implemented for this purpose in several COVID-19–designated hospitals in Thailand. These centers are now also gaining experience in performing daily LUS for early detection of disease progression in COVID-19, with results important for decisions on escalation of care.^7^

One challenge with LUS is defining consistent systems for reporting and interpretation of findings. One frequently used approach is semi-quantification of lung aeration across 12 lung regions into a numerical “global score,” in which the presence of A lines counts as “0,” less or more B lines count as “1” or “2,” and consolidation counts as “3.”^8^ Scores are then summed to a global score that ranges from 0 (i.e., “no abnormalities at all”) to 36 (i.e., “consolidations in all regions”). Global LUS scores combined with SpO_2_/FiO_2_ in a resource-limited setting,^9^ and with PaO_2_/FiO_2_ in a resource-rich setting,^10^ showed good performance for diagnosing acute respiratory distress syndrome (ARDS) and for assessing ARDS severity.^11^ Of note, current global LUS scores do not include pleural thickening and subpleural abnormalities, which seem often present in COVID-19. We should evaluate urgently whether a global LUS score has diagnostic and prognostic value in COVID-19, whether scores correlate with disease severity and CT findings, and how pleural thickening and subpleural abnormalities should be incorporated in the scoring system.

Lung ultrasound could potentially also be an important tool for optimizing invasive ventilation in patients with severe COVID-19.^12^ Although patients with COVID-19 receiving invasive ventilation will often have non-recruitable lung lesions early on, recruitable lesions may develop later in the disease course. In patients with lung lesions that can be opened up, recruitment maneuvers and higher positive end-expiratory pressure (PEEP) may be beneficial.^13^ It is currently not well defined how these recruitable lung areas can be best identified. Comparing the same lung lesion on chest CT scan at different PEEP levels (10 and 20 cm H_2_O) can show whether a lesion becomes aerated (Schultz, personal experience in Amsterdam). However, this procedure is impractical, especially in resource-limited settings. In patients with ARDS, it has been shown that PEEP-induced lung recruitment can be adequately estimated with bedside LUS.^14^ A study to assess LUS as a tool to detect recruitable lung lesions in patients with severe COVID-19 is currently underway in Amsterdam, the Netherlands, and Brussels, Belgium.

In summary, the report from Yasukawa and Taro^6^ shows that LUS is a promising additional lung imaging tool in COVID-19, in particular in settings with limited resources. This easy-to-perform bedside tool could prove to be important in diagnosis, prognostication, and follow-up of patients, and might also enable identification of recruitable lung lesions to guide invasive ventilation.
